# Optimized cloaks made of near-zero materials for different-sized concealed targets

**DOI:** 10.1038/s41598-018-34771-3

**Published:** 2018-11-13

**Authors:** Zhenzhong Yu, Zhong Yang, Yuehong Wang, Haifei Si, Guoshu Zhao

**Affiliations:** 1 School of Intelligence Science and Control Engineering, Jinling Institute of Technology, Nanjing, 211169 China; 2 School of Foreign Language, Nanjing Xiaozhuang University, Nanjing, 211171 China

## Abstract

The optimized cloaking design for conducting cylinders of different sizes is studied based on the Mie scattering theory. We construct a concentric multi-layered cloak made of alternating materials with isotropic dielectrics and epsilon-near-zero (ENZ) material, the thickness of which can be determined through genetic algorithm. As the radius of the conducting cylinder increases, high order scattering contributions are becoming evident, and more layers are needed. The scattering cross sections of three different radii of PEC cylinders are minimized by utilizing different numbers of multi-layers respectively. We find that eight or less optimized layers can cancel most of the scattering from a conducting cylinder with its dimension compared to wavelength, and more effectively when taking the ENZ material as the inner starting shell. The frequency dependence of total scattering is also studied, leading to the result that the bandwidth decreases as the size of concealed PEC cylinder increases. Furthermore, it is shown that the cloaking efficiency is less sensitive to the permittivity and thickness of the ENZ material, due to the small phase variation in the ENZ material. The multi-layered cloak designed for a PEC target can also be used to evidently reduce the scattering of a dielectric core and design a multi-layered elliptical cloak.

## Introduction

During the past decade, there has been a great deal of interest in studying the electromagnetic (EM) invisibility cloak, which can hide objects from EM detection^1–11^. To cancel the EM scattering from a given object, several schemes have been proposed, such as transformation based cloaking^1–7^, plasmonic cloaking due to scattering cancellation^8–10^, and active cloaking^11^, etc. The transformation optical scheme can make a macroscopic object invisible perfectly, but it requires the material with continuously varying inhomogeneous and anisotropic permittivity and permeability with extreme values, which is impossible to fabricate in the ideal scheme and still a formidable task even after some simplified approximations^1–7^. In contrast to the extraordinarily complex material obtained from the coordinate transformation, the scattering cancellation scheme commonly termed as plasmonic cloaking only uses one or two layers of isotropic plasmonic covers to cancel the dipole response of an object^8–10^. However, this cloaking scheme is typically limited to sub-wavelength scales, while for larger object the higher order scattering may make the cloaking performance less effective. In order to facilitate the physical implementation and conceal a moderate-sized object with maximum level of invisibility, several kinds of multi-layered cloaks based on the optimization procedures have been proposed, employing anisotropic metamaterials^12–14^, isotropic ENZ materials^15,16^, and even ordinary dielectrics^17–19^ respectively.

Realization of the two main cloaking schemes above mentioned is usually connected with the design of a material whose permittivity is close to zero, namely ENZ material. With low wave number and negative polarization with respect to vacuum, the ENZ material exhibits some anomalous applications such as squeezing electromagnetic energy through narrow sub-wavelength channels, and shaping the wave fronts parallel to the ENZ exit interface^20^. Recently, it was experimentally demonstrated that the ENZ material can act as a host medium doped by dielectric particles to modify the medium’s effective permeability, independently of the dopants’ position within the host^21^. In the respect of invisibility, an ENZ layer with properly designed thickness can induce an opposite dipole moment between the core and the outer ENZ layer to cloak an object^8^. A double-layer cloak, consisting of an ENZ layer and a dielectric layer, was also reported to provide simultaneously the shielding of the cloaked volume and substantial reduction of the scattering of a moderate-sized target^22^. The inner ENZ layer regarded as a screening shell can vanish the field in the core target, and produce the super-localization effect of the field in the ENZ layer^22^. Moreover, the optimized multi-layered cloak of more than two shells, consisting of layers of dielectric materials and ENZ materials, can provide more degrees of freedom to achieve a quasi-perfect cloaking performance^15^. The ENZ layer in the multi-layered structure plays a role in concentrating the EM power and guiding it propagating around the core^15^. There are two approaches to obtain the ENZ material. It may be found naturally at infrared and visible frequencies, such as noble metals, some semiconductors, and polar dielectrics near their plasma frequency^23^. Alternatively, it can be fabricated artificially at the desired frequency by embedding suitable inclusions in a host medium^24^.

In the previous work, we have found that most of the multipolar scattering terms of a moderate-sized PEC target can be effectively canceled by employing appropriate numbers of covering shells^15^. And, it is more difficult to conceal a large target as more scattering terms are becoming evident. Although some results have been obtained before, there are still several interesting and important questions worth answering, as follows. (I) How many layers are needed to conceal the PEC cylinders with different radii? (II) Which composition order of materials has better performance, considered from several aspects such as total scattering section, bandwidth, influence of losses in the ENZ material, and parameter variation? (III) Which scattering terms are canceled by each layer respectively? (IV) Can the predesigned cloak for PEC target be used to reduce the scattering from a dielectrics, or to design a cloak with another shape? (V) Is it possible to achieve a quasi-perfect cloaking of a dielectric cylinder with moderate size? In this paper, the main objective is to examine the cloaking performance in two kinds of layered structures: the ENZ-dielectric-ENZ… multi-layered structure (Z-D pattern) and the dielectric-ENZ-dielectric… multi-layered structure (D-Z pattern). Considered from different aspects, the multi-layered cloak under Z-D pattern has better comprehensive performance. We find that most of the scattering from a conducting cylinder with its dimension compared to wavelength can be canceled by using eight or less optimized layers. The multi-layered cloak designed for a PEC target can also be used to evidently reduce the scattering of a dielectric core in most cases. Besides the circular cloak, it is also possible to design the multi-layered elliptical cloak, in which the thickness of the each covering shell is the same as that of the cloak once designed for the PEC cylinder.

## Results

**Figure 1:**
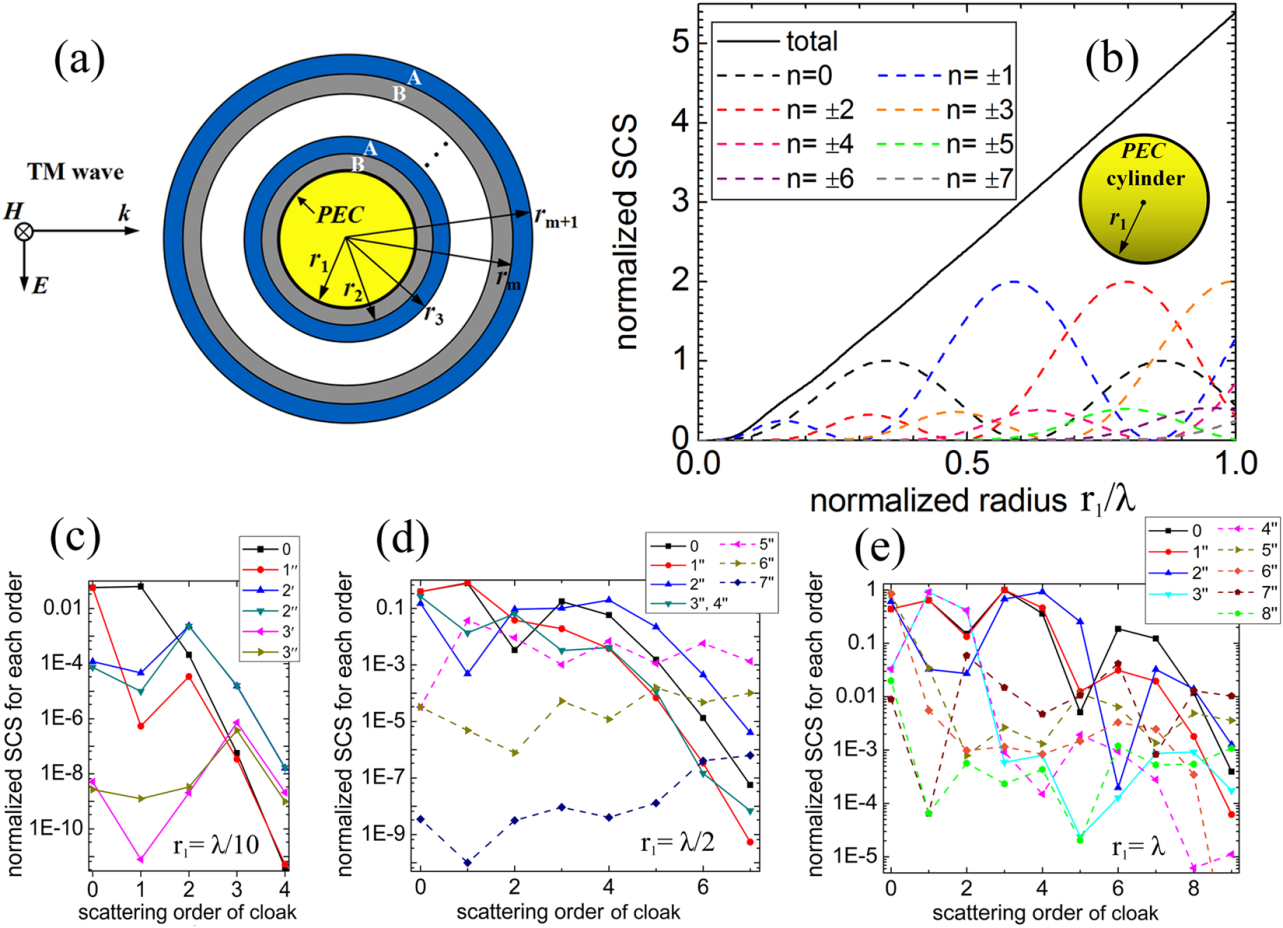
(**a**) TM plane wave is normally impinging on an infinitely long PEC cylinder, coated by m shells of alternating material A and material B. The radius of the PEC cylinder is *a* = *r*_1_, and the radii of the covering shells are *r*_*i*_ (*i* = 2, …, *m* + 1). (**b**) Total SCS of a bare PEC cylinder at different radii and its contributions from the first seven modes. (**c**) Each scattering order contribution of the optimized multi-layered cloaks with inner PEC cylinder *r*_1_ = λ/10, including the Z-D pattern and the D-Z pattern. (**d**,**e**) Each scattering order contribution of the cloaks with PEC cylinders *r*_1_ = λ/2 and *r*_1_ = λ respectively, for simplicity, only including the Z-D pattern.

First, we assume that a TM polarized plane wave with unite amplitude is impinging upon an infinitely long PEC cylinder, coated by m-layers of alternating material A and material B as depicted in Fig. 1(a). The radii of the m-layer cylindrical structure are denoted as *r*_*i*_ (*i* = 1, 2, …, *m* + 1). The form of time-harmonic variation is represented by *e*^*jωt*^. According to the Mie theory^25^, the total SCS of the multilayers is determined by all the scattering coefficients

1 $${C}_{scs}=\frac{4}{{k}_{0}}\sum _{n=-{\rm{\infty }}}^{{\rm{\infty }}}{|{c}_{n}|}^{2}$$

Where ${k}_{0}=\omega \sqrt{{\mu }_{0}{\varepsilon }_{0}}$ is the free space wave number.

To compare the scattering of different multi-layered structures, we introduce the normalized SCS, defined by the ratio of total SCS to 4/*k*_0_. This normalized SCS can be decomposited into the summation of all the single mode SCSs as

2 $${C}_{nscs}=\sum _{n=-{\rm{\infty }}}^{{\rm{\infty }}}{C}_{nscs}^{n}=\sum _{n=-{\rm{\infty }}}^{{\rm{\infty }}}{|{c}_{n}|}^{2}$$

In this study, we aim to reduce the scattering of PEC cylinders with different radii. For the optimized cloaking design of the multi-layered structure, genetic algorithm (GA)^26^ is used to minimize the total SCS by searching the optimal permittivity and the thickness of the covering layers. Figure 1(b) shows the normalized total SCS for the bare PEC cylinder as a function of radius, as well as the contributions from different single modes, that is monopole (*n* = 0), dipole (*n* = ±1), quadrupole (*n* = ±2) and higher scattering modes. When the dimension of the PEC cylinder is much smaller than the working wavelength (${r}_{1} < \lambda /10$), namely quasistatic approximation, the scattering properties are dominated by the *n* = 0 and *n* = ±1 terms. For the object dimension comparable to wavelength, the higher order scattering contributions are becoming apparent. As the normalized radius is approaching one, all first seven terms should be considered.

To simplify the practical realization, we consider a multi-layered cloak consisting of two kinds of alternating material A and B, one normal dielectric and the other ENZ medium. In the optimization process of this study, we find that GA is inclined to select two kinds of materials with large dielectric constants difference. Therefore, we choose the near-zero material with *ε*_*z*_ = 0.1, and the normal dielectric with a relative large permittivity *ε*_*d*_ = 12.08, which can be obtained by the commonly used material silica at wavelength *λ* = 1550 nm^27^. The near-zero material with permittivity much less than unity can be available at infrared and visible range, for example, the noble metals, polar dielectrics or some semiconductors usually with a dispersion property described by the Drude models^28^. Table 1 shows the normalized SCSs of optimized multi-layered shells covering the PEC cylinder with three different radii *r*_1_ = *λ*/10, *λ*/2 and *λ*, respectively. The superscript ‘′’ indicates the inner starting shell is the dielectric material corresponding to the alternating layers of dielectric and ENZ material (D-Z pattern), while superscript ‘″’ indicates the innermost shell is the ENZ material (Z-D pattern). As to the PEC cylinder with radius *r*_1_ = *λ*/10, the normalized SCS is 0.183, mainly contributed from the monopole and dipole scattering modes. Seen from the configuration 1′ and 1″ in the table, when covered by one-layer material, only ENZ material can reduce the SCS. This physical mechanism for reducing the total scattering is that the overall dipole moment is canceled because induced polarization in the ENZ material is oppositely oriented with respect to that in the PEC cylinder^8^. In the quasistatic case, the parameters for this one-shell cylindrical structure can be calculated analytically from the dipolar transparency condition $\gamma =\frac{{r}_{1}}{{r}_{2}}=\sqrt{\frac{{\varepsilon }_{0}-{\varepsilon }_{z}}{{\varepsilon }_{0}+{\varepsilon }_{z}}}$, where γ is the ratio of core-shell radii and the formula is for the case of TM polarization with magnetic field parallel with the cylinder axis^8^. If we take ε_z_ = 0.1 in the above formula, the shell radius can be calculated as *r*_2_ = 1.106*r*_1_ = 0.1106*λ*, while the corresponding optimized value is *r*_2_ = 0.1096*λ*. This little deviation is because that rency condition only considers the dipolar scattering term, but ignores the monopole term. With the increase of layer number, the scattering decreases gradually and using only three layers can achieve quasi-perfect invisibility with normalized SCS equal to 7.8 × 10^−7^. With the increase of PEC radius, for example *r*_1_ = *λ*/2 or *λ*, more optimized shells are utilized to reduce the total SCS since more scattering terms activate. As can be seen from the table, taking the ENZ material as the inner starting shell is more effective in reducing the scattering. Despite of the general trend that more optimized layers result in lower scattering, there also exists an abnormal situation in the optimization that adding more layers does not further reduce the SCS, for example λ/2 PEC cylinder covered by 3-layer and 4-layer cloak with same SCS. For the λ/2 PEC cylinder, the total SCS decreases to an extremely low level with 7 layers. Even with a larger radius (*r*_1_ = *λ*), 163 times reduction of total SCS can be achieved by using 8 layers. The cloaking mechanism for the plasmonic multilayer covering a large target is as follows. When the dimension of the target is comparable with the wavelength, the incident field is not uniform across the target, and the multipolar moments are becoming apparent. By using a properly designed multi-layer consisting of ENZ and dielectric materials, oppositely-signed multipolar moments can be induced to cancel the ones produced by the big target, and thus providing a cloaking cover. Moreover, we have found that the dielectric layers sandwiched by the double-zero material with the newly optimized thickness can also reduce the scattering of the PEC cylinder greatly. This cloak, in which the double-zero materials can be realized by the dielectric photonic crystals^29^, will be further studied in the future work.

**Table 1:** Optimized results of the cylindrical multi-layered structure for reducing the scattering of PEC cylinder.

Config.	SCS	r_1_	r_2_	r_3_	r_4_	r_5_	r_6_	r_7_	r_8_	r_9_
0	0.183	λ/10								
1′	0.183	λ/10	λ/10							
1″	0.057	λ/10	0.1096λ							
2′	0.0049	λ/10	0.1593λ	0.1803λ						
2″	0.0047	λ/10	0.1640λ	0.1807λ						
3′	1.5 × 10^−6^	λ/10	0.15211λ	0.17492λ	0.18027λ					
3″	7.8 × 10^−7^	λ/10	0.14251λ	0.16766λ	0.17359λ					
0	2.404	λ/2								
1′	2.404	λ/2	λ/2							
1″	2.106	λ/2	0.5056λ							
2′	1.681	λ/2	0.5629λ	0.5939λ						
2″	0.975	λ/2	0.5257λ	0.5976λ						
3′	0.823	λ/2	0.5515λ	0.5775λ	0.6015λ					
3″	0.432	λ/2	0.5146λ	0.5890λ	0.5971λ					
4′	0.432	λ/2	λ/2	0.5146λ	0.5890λ	0.5971λ				
4″	0.432	λ/2	0.5146λ	0.5890λ	0.5971λ	0.5971λ				
5′	0.384	λ/2	0.5126λ	0.6294λ	0.8933λ	0.9135λ	0.9481λ			
5″	0.125	λ/2	0.7269λ	0.7631λ	0.7788λ	0.8933λ	0.9117λ			
6′	0.043	λ/2	0.7810λ	0.8268λ	0.8943λ	0.9398λ	1.0640λ	1.0836λ		
6″	8.2 × 10^−4^	λ/2	0.5353λ	0.5873λ	0.6425λ	0.9152λ	0.9338λ	0.9421λ		
7′	2.3 × 10^−5^	λ/2	0.63395λ	0.76704λ	0.80331λ	0.83778λ	0.95775λ	0.97677λ	0.98330λ	
7″	3.2 × 10^−6^	λ/2	0.53564λ	0.58564λ	0.64566λ	0.90884λ	0.91753λ	0.93754λ	0.94395λ	
0	5.391	λ								
1′	5.391	λ	λ							
1″	5.041	λ	1.0038λ							
2′	4.326	λ	1.065λ	1.098λ						
2″	4.511	λ	1.060λ	1.098λ						
3′	3.101	λ	1.055λ	1.080λ	1.114λ					
3″	2.694	λ	1.018λ	1.127λ	1.142λ					
4′	2.664	λ	1.0084λ	1.028λ	1.135λ	1.148λ				
4″	2.670	λ	1.019λ	1.127λ	1.141λ	1.143λ				
5′	2.293	λ	1.064λ	1.215λ	1.338λ	1.357λ	1.387λ			
5″	0.961	λ	1.025λ	1.111λ	1.161λ	1.287λ	1.307λ			
6′	0.943	λ	1.016λ	1.046λ	1.117λ	1.156λ	1.280λ	1.299λ		
6″	0.896	λ	1.023λ	1.104λ	1.162λ	1.287λ	1.308λ	1.316λ		
7′	0.896	λ	λ	1.023λ	1.104λ	1.162λ	1.287λ	1.308λ	1.316λ	
7″	0.390	λ	1.173λ	1.208λ	1.248λ	1.371λ	1.452λ	1.584λ	1.610λ	
8′	0.390	λ	λ	1.173λ	1.208λ	1.248λ	1.371λ	1.452λ	1.584λ	1.610λ
8″	0.036	λ	1.0392λ	1.0956λ	1.1904λ	1.3226λ	1.4016λ	1.6778λ	1.6968λ	1.7083λ

Three different sizes of bare PEC cylinders with radii r_1_ = λ/10, λ/2 and λ are optimized by covering multi-layers of alternating ENZ material and dielectric material. The superscript ‘′’ indicates the inner starting shell is the dielectric material, while superscript ‘″’ indicates the innermost shell is the ENZ material.

The scattering multipoles of the multi-layered cloaks with inner PEC cylinder *r*_1_ = λ/10, λ/2 and λ respectively have also been calculated. As to the small PEC cylinder *r*_1_ = *λ*/10, the cloak constructed by only one ENZ layer, denoted by 1″, mainly cancels the dipole scattering term n = 1, shown in Fig. 1(c). The two-layer cloak, including the D-Z pattern 2′ and the Z-D pattern 2″, can suppress both of the dipole term and the monopole term. The cloak with three layers, the first three scattering terms are dramatically decreased. When the radius is increased to λ/2, high order scattering terms are significantly increased, for instance *n* = 3 and *n* = 4, shown in Fig. 1(d). We can find that the one-ENZ-cloak 1″ can not suppress the dipole and the monopole scattering terms of λ/2 PEC cylinder, and the multi-layered cloaks with two and three layers mainly cancel the dipole term. Only when the layer number is more than four, can the dominant scattering terms be evidently decreased. For the PEC cylinder with *r*_1_ = λ, more scattering terms become prominent, i.e. the first eight scattering terms except *n* = 5, shown in Fig. 1(e). The one-ENZ-cloak 1″ tends to decrease the higher order scattering terms, but has almost no effect on the first six scattering terms. It can be seen that the cloaks from 2″ to 6″ can only suppress some of the prominent scattering terms, whereas these scattering terms can be greatly decreased by the cloaks 7″ and 8″.

**Figure 2:**
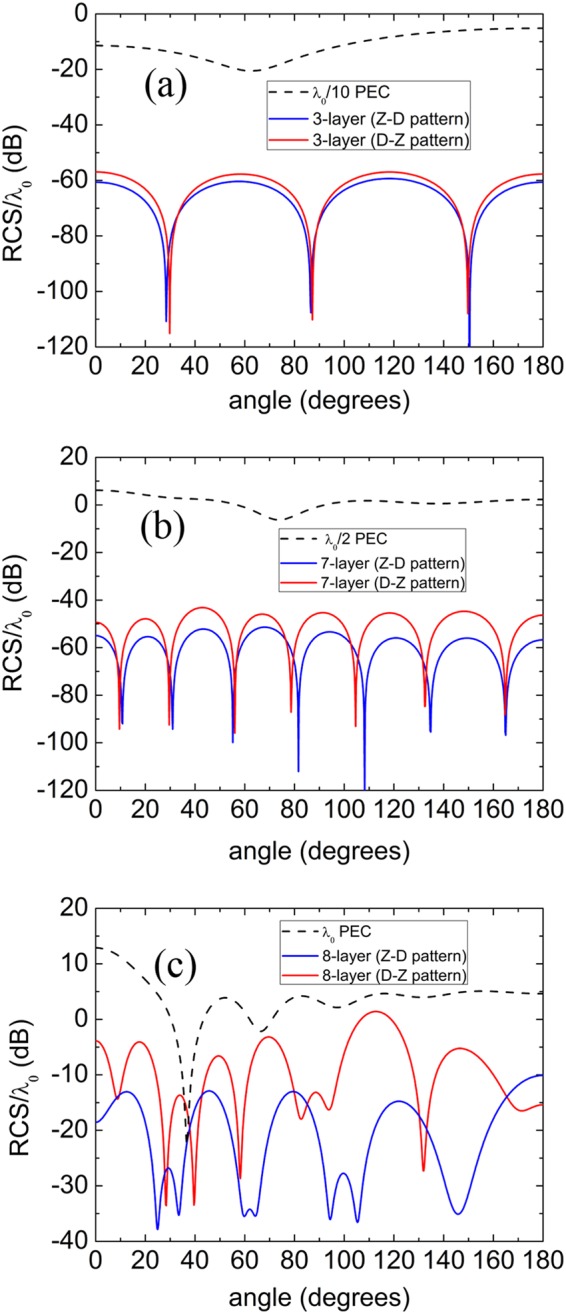
The scattering width normalized by wavelength for the PEC cylinder (dashed lines), the optimized shells with D-Z pattern (solid red lines), and the optimized shells with Z-D pattern (solid blue lines). Three PEC cylinders with radius *r*_1_ = *λ*/10 (**a**), *r*_1_ = *λ*/2 (**b**) and *r*_1_ = *λ* (**c**) are considered.

Although the total SCSs for PEC cylinders, showing a general scattering performance in all directions, can drop to a low level, the calculation of bistatic scattering width is still necessary for us to understand exactly the far-field scattering distribution. In Fig. 2(a), the scattering performance of the optimized 3-layer under the Z-D pattern has similar scattering diagram to that of D-Z pattern, and the scattering width of both patterns is 40 dB lower than that of the bare PEC cylinder at most of the angles. As the radius of the PEC cylinder increases, the forward scattering of the bare PEC seems more serious than other directions, for example *λ*/2 PEC cylinder marked by dashed line in Fig. 2(b). As the radius further increased to λ, the forward scattering is more prominent with normalized RCS 12.5 dB, as shown in Fig. 2(c). We notice that the scattering width of the optimized 8-layer under the Z-D pattern is greatly reduced, especially in the forward direction with a reduction of 30 dB.

## Discussion

**Figure 3:**
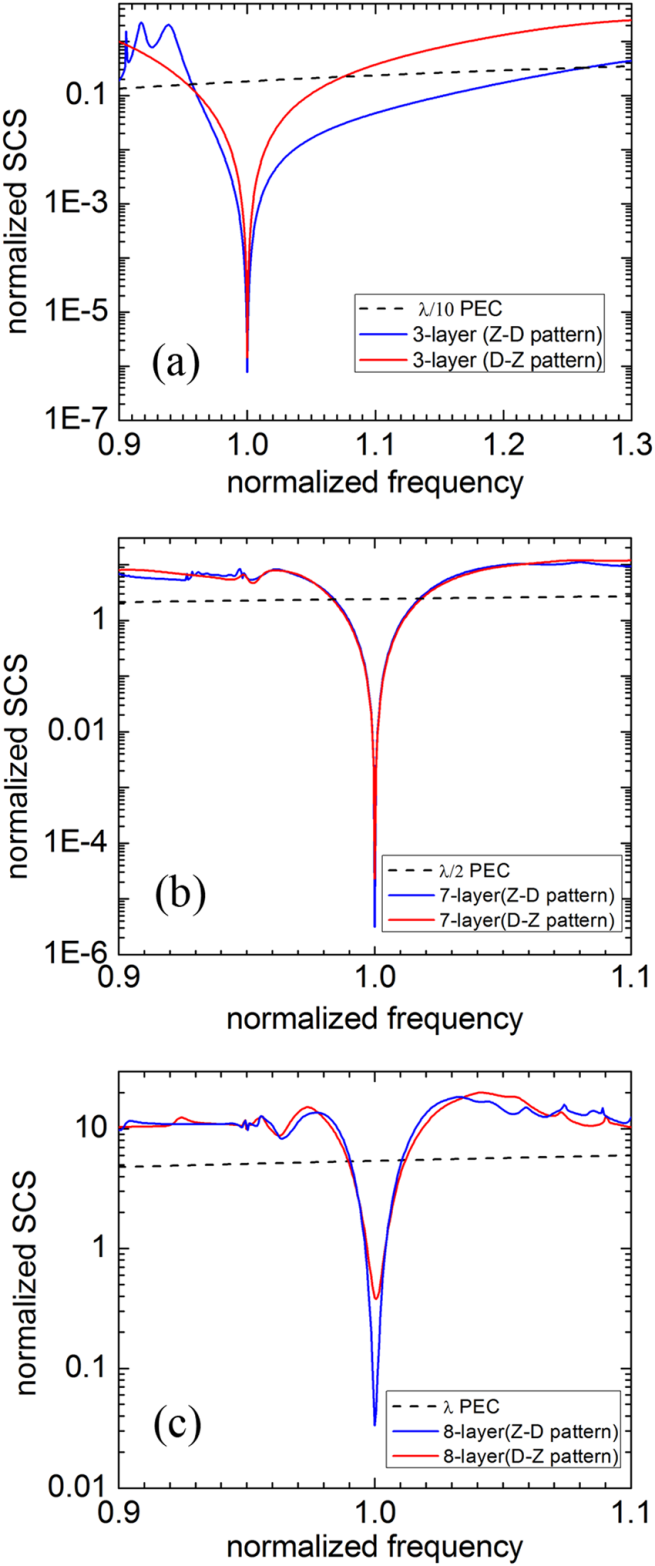
The normalized total SCSs as a function of normalized frequency *f*/*f*_0_ for the optimized multi-layered shells covering the PEC cylinders with three different radii *r*_1_ = *λ*/10 (**a**), *λ*/2 (**b**) and *λ* (**c**), respectively. The total SCSs of PEC cylinders are also plotted in the each figure for comparison (black dashed line).

The dielectric constant of the near-zero material always disperses heavily as frequency changing and is supposed to follow the Drude model with ${\varepsilon }_{z}(f)={\varepsilon }_{0}(1-{f}_{p}^{2}/{f}^{2})$, where *f*_*p*_ is the plasma frequency. We choose the design frequency *f*_0_ = 1.054 *f*_*p*_ in the Drude model to ensure the dielectric constant ${\varepsilon }_{z}({f}_{0})=0.1{\varepsilon }_{0}$. Figure 3 shows the normalized SCS as a function of normalized frequency *f*/*f*_0_ for the bare PEC cylinder (dashed line), and for the best optimized shells including the Z-D pattern (blue solid line) and D-Z pattern (red solid line). It can be seen that the SCS of the optimized cylindrical multilayer allows a drastic reduction of the scattering at the central frequency and increases rapidly when the working frequency deviates from it. The invisibility bandwidth, in which the total scattering of the optimized multilayered cloak is smaller than that of bare PEC cylinder, described by a relative frequency range Δ*f*/*f*_0_ is considered as follows. For the small PEC cylinder (r_1_ = *λ*/10), the 3-layer cloak under Z-D pattern has a broad bandwidth about 30%. However, the bandwidth for the optimized 7-layer cloak (*r*_1_ = *λ*/2) of both patterns is only about 3.4%, and the bandwidth for the 8-layer cloak (r_1_ = *λ*) is about 2.1%. This result indicates that the bandwidth decreases as the size of concealed PEC cylinder increases. Moreover, compared to other optimized schemes including the multilayered ordinary-dielectrics cloak^18^ and the multilayered anisotropic-metamaterial cloak^14^, the bandwidth of the cloak made of ENZ materials proposed in this paper has similar performance.

**Figure 4:**
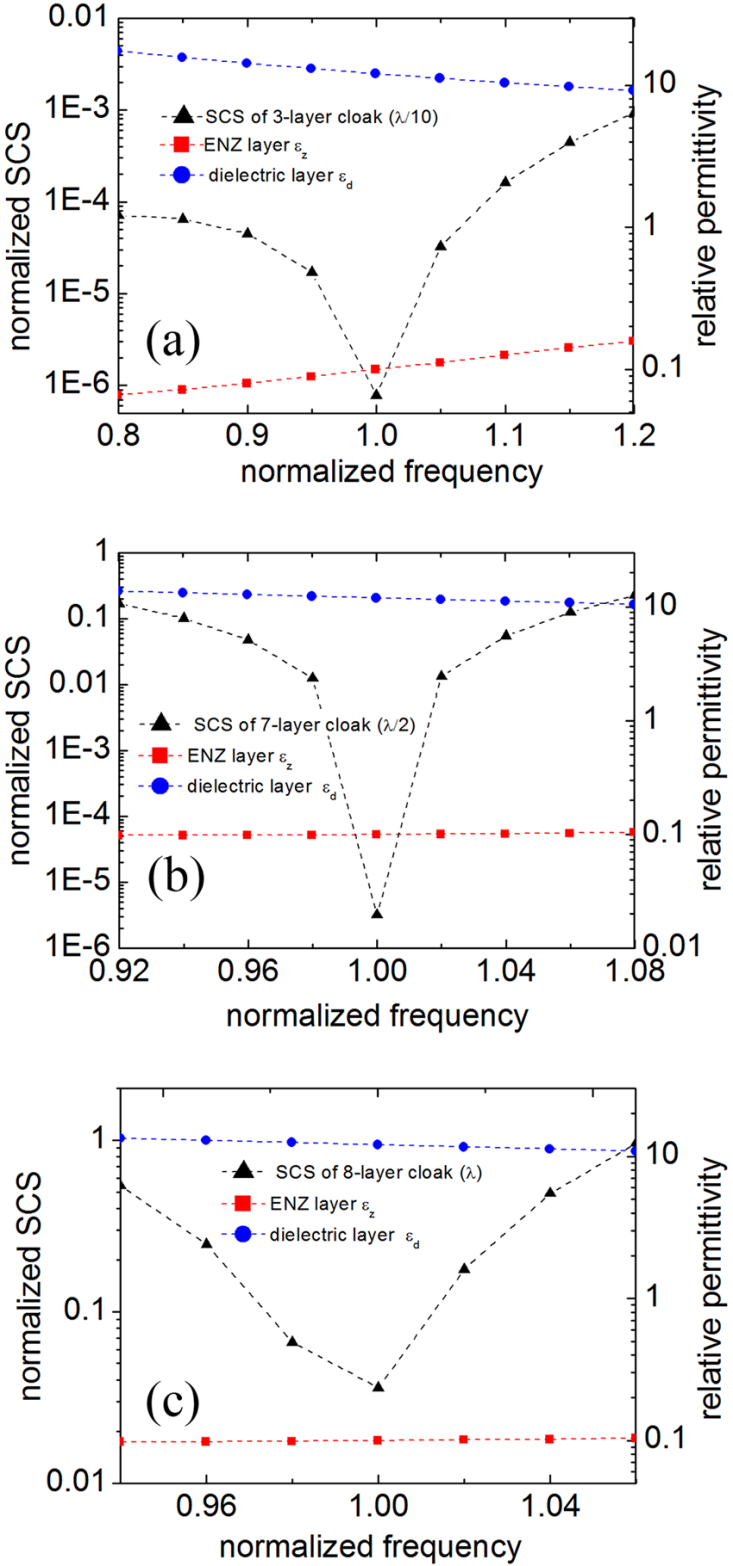
The geometric structures of the multi-layered cloaks under Z-D pattern are optimized at the central frequency *f*_0._ We fix the thickness and change the permittivity to achieve better cloaking performance in the vicinity of the central frequency. The inner PEC cylinders with three different radii *r*_1_ = λ/10 (**a**), λ/2 (**b**) and λ (**c**), are considered respectively. The normalized total SCSs for the multi-layered shells are denoted by the left ordinate. The relative permittivities of the dielectric layer and the ENZ layer are denoted by the right ordinate.

Considering the dispersion property of the covering materials, we may wonder whether it is possible to optimize the bandwidth by searching the material parameters of the multi-layered cloak whose geometric structures are designed at the central frequency. For the 3-layer cloak with inner small PEC cylinder (*r*_1_ = *λ*/10), the normalized SCS rapidly increases when the working frequency deviates the central frequency, shown in Fig. 4(a). We also see that the permittivity of the ENZ layer increases with the increase of frequency, exhibiting normal dispersion. However, the dielectric layer exhibits anomalous dispersion behavior in the considered frequency range. Although the SCS is sensitive to the frequency, it is still much smaller than the SCS of the bare PEC cylinder, because optimization of the two parameters can reduce apparently the SCS of the small PEC target. As to the larger PEC cylinder *r*_1_ = *λ*/2 and *λ* shown in Fig. 4(b,c), the SCSs increase to a considerable level in the vicinity of the central frequency, and the two materials seem to have very weak dispersion properties. Therefore, it is hardly possible to design a wideband multi-layered cloak even by employing the anomalous dispersion material, especially for the larger PEC target due to the small numbers of optimized parameters.

**Figure 5:**
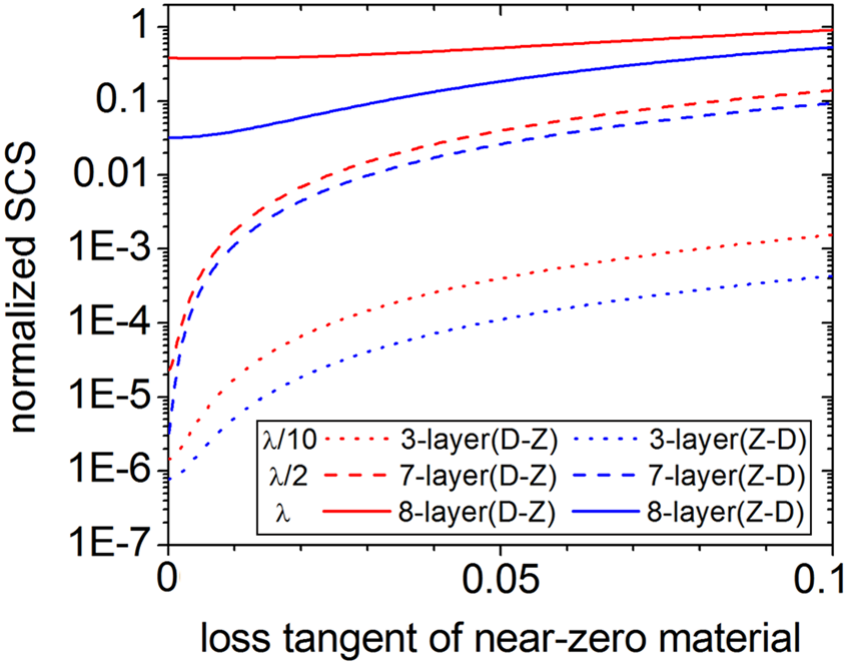
The normalized SCSs of the optimized multi-layered cloak with different loss tangent of near-zero material. Three different concealed PEC cylinders with radii *r*_1_ = λ/10 (dotted lines), λ/2 (dashed lines) and λ (solid lines) are considered. The SCSs of D-Z pattern (red lines) and Z-D pattern (blue lines) are both plotted in the figure for comparison.

We have also investigated the influence of losses in the near-zero material on the invisibility performance. In Fig. 5, the normalized SCSs of the optimized multi-layers covering the inner PEC cylinders with three radii *λ*/10, *λ*/2 and *λ* have been calculated by introducing different loss tangents $\tan \,\delta =\frac{{{\varepsilon }^{{\rm{^{\prime} }}{\rm{^{\prime} }}}}_{z}}{{{\varepsilon }^{{\rm{^{\prime} }}}}_{z}}$, where ε_z_′ and ε_z_″ are the real part and imaginary part of dielectric constant of the near-zero material. It can be seen that all the SCSs increase monotonously with the increase of loss tangent, except for the condition that the SCS of the 8-layer cloak under the D-Z pattern (solid red line) will be a minimum when the loss tangent is around 0.006. And this is in accordance with the conclusion obtained from the optimized ordinary-dielectrics cloak^18^ that the SCS of large target may get a minimum at a certain value of loss tangent. We also find that the SCSs under Z-D pattern are always lower than those under the D-Z pattern with the same concealed target. When the loss tangent is 0.1, all SCSs of the optimized cloak under both patterns are much lower than those of the corresponding bare PEC cylinders. For example, the SCS of the 8-layer cloak under Z-D pattern (solid blue line) is about 10% the value of bare PEC cylinder with radius *r*_1_ = *λ*, the 7-layer cloak (dashed blue line) is about 4% the value of the λ/2 PEC cylinder, and the 3-layer cloak (dotted blue line) is only about 2% the value of the *λ*/10 PEC cylinder. This indicates that with the same material loss in the ENZ material, the smaller the PEC target is, the smaller the ratio of the SCS for the optimized cloak to that for the bare PEC target will be.

**Figure 6:**
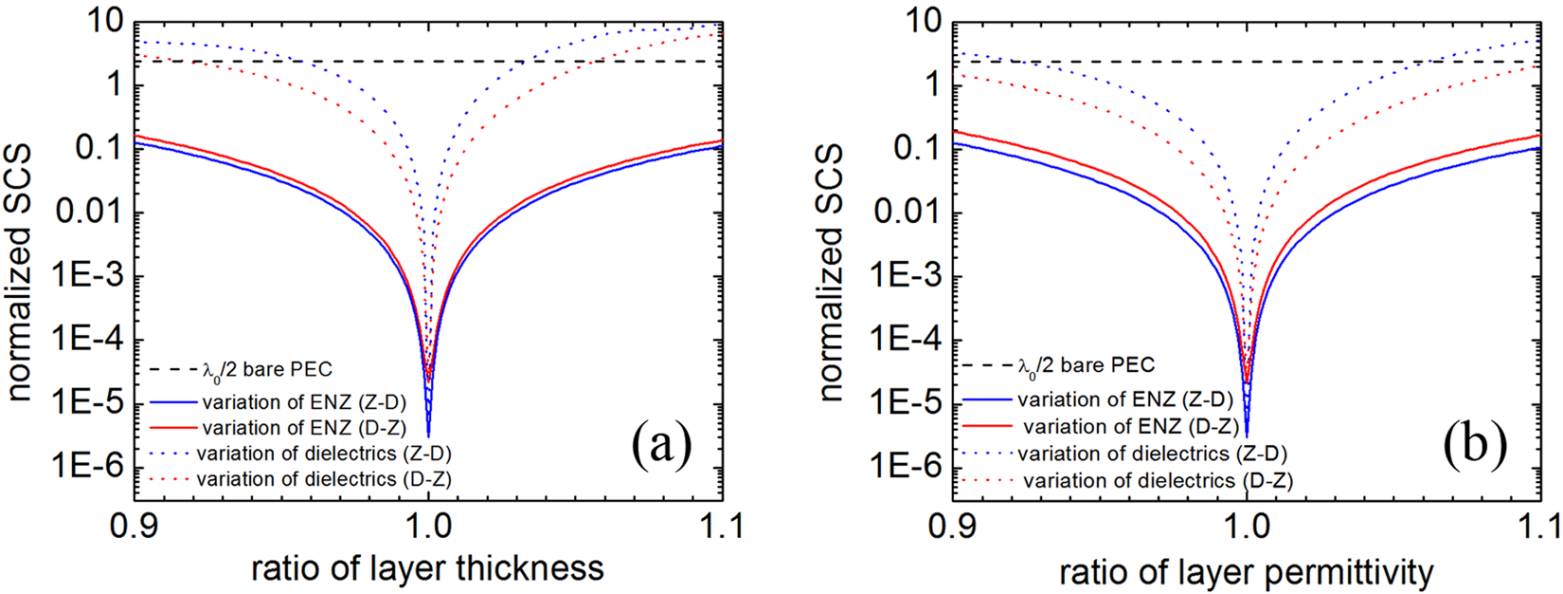
(**a**) Dependence of the normalized SCSs on the layer thickness for the optimized 7-layer cloak with inner λ_0_/2 PEC core. (**b**) Dependence of the normalized SCSs on the layer permittivity for the optimized 7-layer cloak with inner λ_0_/2 PEC core. The SCSs in different cases are plotted in figure (**a**,**b**) for comparison: λ_0_/2 bare PEC (dashed black lines), variation of only ENZ layers under Z-D pattern (solid blue lines), variation of only ENZ layers under D-Z pattern (solid red lines), variation of only dielectric layers under Z-D pattern (dotted blue lines), and variation of only dielectric layers under D-Z pattern (dotted red lines).

Furthermore, sensitivity of the optimized cloak to the thickness tolerances of ENZ and dielectric layers is studied. Here, for simplicity, we only present the influence of thickness variation for the 7-layer optimized cloak with inner *λ*/2 PEC core, shown in Fig. 6(a). It can be seen that the cloaking performance is less sensitive to the ENZ thickness under both D-Z and Z-D patterns. Although the sensitivity increases when only the dielectric-layer thickness is varied, there is still relative thickness variation region of about 13.5% for the D-Z pattern and 7.5% for the Z-D pattern, in which the SCS of the deviated cloak is smaller than that of bare PEC cylinder. Other optimized cloaks for *λ*/10 and *λ* PEC cylinders have also been studied from the above two aspects and tested with similar characteristics yet not shown in the paper. Moreover, we have also studied how much the variation of the permittivities of ENZ and dielectric layers influences the total scattering. Figure 6(b) shows that cloaking performance is also less sensitive to the ENZ permittivity under both D-Z and Z-D patterns. The above results indicate that the total scattering of the optimized cloak is mainly controlled by the thickness and permittivity of the dielectric layer. The mechanism is that the phase variation is very small when the thickness and permittivity of the ENZ material are slightly tuned. Another feature of the ENZ material such as squeezing electromagnetic energy has also been reported in our previous cloak research^15^.

**Figure 7:**
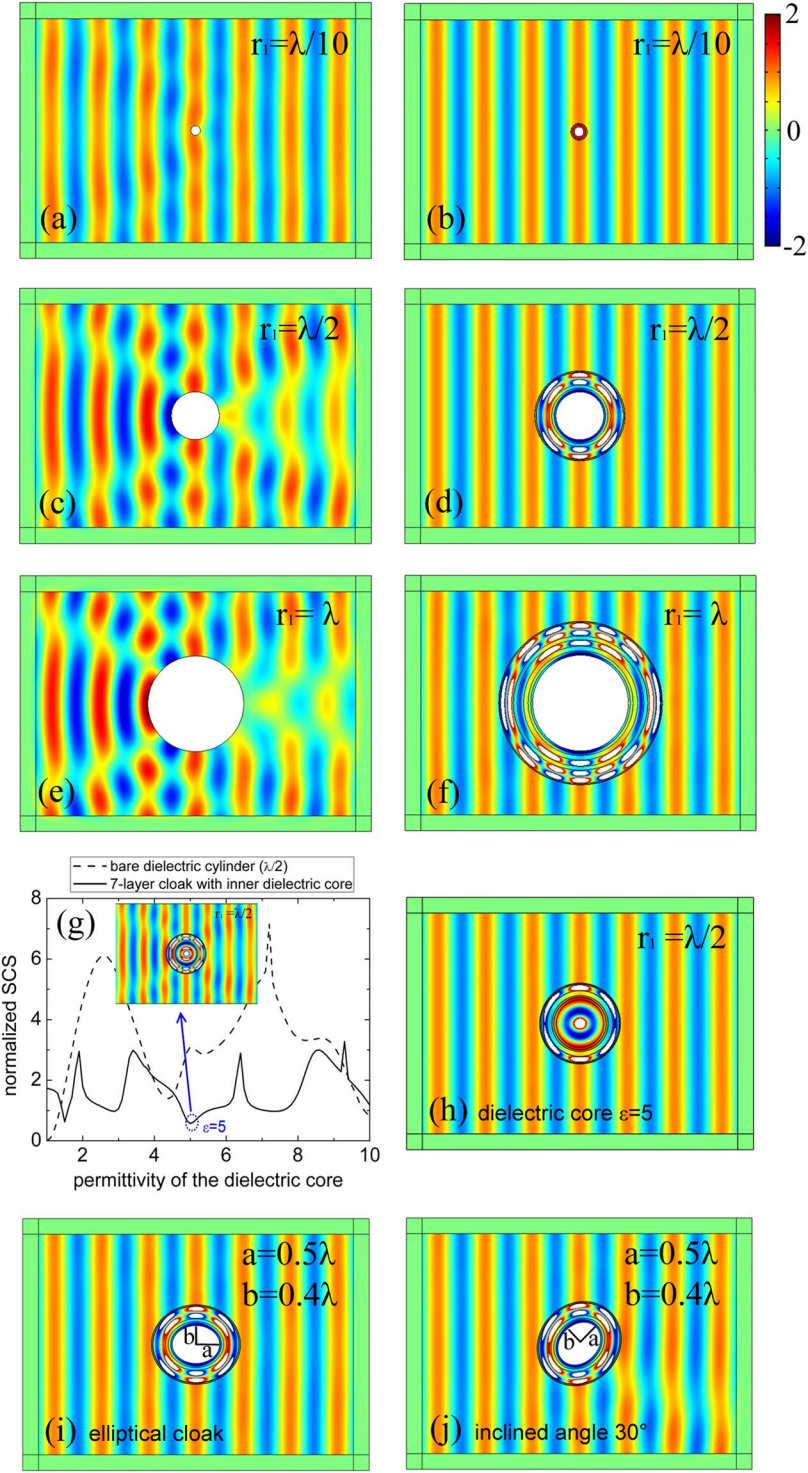
(**a**,**b**) Magnetic field distribution of the bare PEC cylinder r_1_ = *λ*/10 and the optimized multi-layered cloak 3″. (**c**,**d**) Field distribution of the bare PEC cylinder r_1_ = *λ*/2 and the optimized cloak 7″. (**e**,**f**) Field distribution of bare PEC cylinder r_1_ = λ and the optimized cloak 8″. (**g**) The cloak 7″ once designed for λ/2 PEC cylinder is used to cloak a dielectric core. The black solid line represents the SCS of 7-layer cloak at different dielectric permittivities, and the dashed line is the SCS of the bare dielectric cylinder (r_1_ = λ/2). The inset is the full wave simulation of the cloak 7″ with inner dielectric cylinder ε = 5. (**h**) The field distribution for a redesigned 6-layer cloak with inner dielectric cylinder ε = 5. (**i**) A 7-layer elliptical cloak covers an PEC ellipse with the semi-major axis a = 0.5λ and the semi-minor axis b = 0.4λ. The thickness of the each covering shell is the same as that of the cloak 7″ once designed for λ/2 PEC cylinder. (**j**) Field distribution of the 7-layer elliptical cloak with an inclined angle 30°.

To verify the optimization results, full-wave EM simulation are carried out to visualize the performance of the optimized multilayered cloaks in comparison with that of the bare PEC cylinders. In Fig. 7(a–f), the field distribution around the small bare PEC target (*r*_1_ = *λ*/10) is little perturbed, whereas the PEC cylinder with radius *r*_1_ = *λ* can induce a remarkable scattering and an evident shadow behind the cylinder. In contrast, the optimized multi-layered cloaks in the three different cases provide good cloaking performance. We have also calculated the SCS of the multi-layered cloak, in which the inner PEC cylinder is replaced by a dielectric one. To simplify, here we only present the influence of dielectric core variation for the 7-layer optimized cloak 7″ with inner radius *r*_1_ = λ/2, shown in Fig. 7(g). The SCS of the multilayered cloak with different dielectric cores (solid line) and the SCS of bare dielectric cylinders (dashed line) have been plotted. We can see that scattering of the multilayered cloak with dielectric core is much smaller than that of bare dielectric core in most cases. Therefore, the predesigned shells can achieve the multi-target cloaking. The inset in Fig. 7(g) illustrates the field distribution of the cloak 7″ with inner dielectric cylinder *ε* = 5, exhibiting a little perturbation. In order to realize a quasi-perfect invisibility for the dielectrics *ε* = 5, we have redesigned a 6-layer cloak under Z-D pattern, with geometric parameters as *r*_1_ = λ/2, *r*_2_ = 0.5563λ, *r*_3_ = 0.5925λ, *r*_4_ = 0.6962*λ*, *r*_5_ = 0.8221λ, *r*_6_ = 0.8442λ, *r*_7_ = 0.853λ. The field distribution outside the cloak is almost no disturbance, shown in Fig. 7(h) and the total normalized SCS is extremely low with the value 4.6 × 10^−4^. Besides the circular cloak, it is also possible to design the multi-layered cloak with another shape. We have designed a 7-layer elliptical cloak covering an PEC ellipse with the semi-major axis *a* = 0.5λ and the semi-minor axis *b* = 0.4λ. The thickness of each covering shell is the same as that of the cloak 7″ once designed for the λ/2 PEC cylinder. The field simulations have validated the cloaking performance at different incident angles, shown in figure (i)(j).

In conclusion, we present an optimization method to design different non-magnetic cylindrical cloaks constructed by alternating layers of isotropic dielectrics and ENZ materials. Through genetic algorithm, the total SCSs of three conducting cylinders with radii *λ*/10, *λ*/2 and *λ* are minimized respectively by covering layers of different numbers. When the radius is *λ*/10, two or three layers can achieve good cloaking performance. As the radius increases, high order scattering contributions are evident, and more layers are needed. When the radius equals wavelength, 163-fold reduction of total SCS can be achieved by employing 8 layers. It is shown that an optimized cloak composed of eight or less layers can suppress the scattering of a conducting cylinder (radius *r*_1_ < *λ*) to a very low level, and more effectively when taking the ENZ material as the inner starting shell. We demonstrate that the cloaking bandwidth of the optimized cloak decreases as the size of concealed PEC target increases. It is almost impossible to design a wideband multi-layered cloak with good invisibility even by employing the anomalous dispersion material, especially for the larger PEC target. We also demonstrate that with the same material loss in the ENZ material, the smaller PEC target is, the smaller SCS for the optimized cloak will be comparing to that for the corresponding bare PEC target. Furthermore, the cloaking efficiency of the optimized multialyer is less sensitive to the permittivity and thickness of the ENZ material, due to the small phase variation in the ENZ material. The multi-layered cloak designed for a PEC target can also be used to evidently reduce the scattering of a dielectric core and design a multi-layered elliptical cloak. The optimization method used in this paper will also be efficient for optimizing superscattering, and other scattering responses of the multi-layered structure.

## Methods

We use genetic algorithm (GA) to optimize the optical properties of cylindrical multi-layered structure^26^. GA is a robust, stochastic search method based on the concept of natural selection and evolution, which is particularly effective when the goal is to find a global maximum or minimum in a high-dimension and complicated function domain. The optimization process is implemented via the Genetic Algorithm Toolbox of MATLAB. Here, the initial population is randomly generated individuals within a constrained domain which is considered from the practical consideration. Three main operations, selection crossover and mutation, are used at each iteration step to create the next generation with more fitter individuals from the current generation. This process is repeated until the algorithm terminates when either a maximum number of generations has been produced, or a satisfactory fitness level has been reached for the population. For the optimized cloaking design of the cylindrical concentric multi-layered structure, GA is used to minimize the total SCS by searching the optimal permittivity and the thickness of the covering layers.
